# A Review of Nervonic Acid Production in Plants: Prospects for the Genetic Engineering of High Nervonic Acid Cultivars Plants

**DOI:** 10.3389/fpls.2021.626625

**Published:** 2021-03-05

**Authors:** Fang Liu, Pandi Wang, Xiaojuan Xiong, Xinhua Zeng, Xiaobo Zhang, Gang Wu

**Affiliations:** ^1^Key Laboratory of Biology and Genetic Improvement of Oil Crops, Ministry of Agriculture and Rural Affairs, Oil Crops Research Institute, Chinese Academy of Agricultural Sciences, Wuhan, China; ^2^Life Science and Technology Center, China National Seed Group Co. Ltd., Wuhan, China

**Keywords:** nervonic acid, production, plant source, 3-ketoacyl-CoA synthase, genetic engineering, oil crops

## Abstract

Nervonic acid (NA) is a very-long-chain monounsaturated fatty acid that plays crucial roles in brain development and has attracted widespread research interest. The markets encouraged the development of a refined, NA-enriched plant oil as feedstocks for the needed further studies of NA biological functions to the end commercial application. Plant seed oils offer a renewable and environmentally friendly source of NA, but their industrial production is presently hindered by various factors. This review focuses on the NA biosynthesis and assembly, NA resources from plants, and the genetic engineering of NA biosynthesis in oil crops, discusses the factors that affect NA production in genetically engineered oil crops, and provides prospects for the application of NA and prospective trends in the engineering of NA. This review emphasizes the progress made toward various NA-related topics and explores the limitations and trends, thereby providing integrated and comprehensive insight into the nature of NA production mechanisms during genetic engineering. Furthermore, this report supports further work involving the manipulation of NA production through transgenic technologies and molecular breeding for the enhancement of crop nutritional quality or creation of plant biochemical factories to produce NA for use in nutraceutical, pharmaceutical, and chemical industries.

## Highlights

This review presents various nervonic acid production related topics in plants, highlighting the factors that affect NA production in genetically engineered oil crops and provides prospects for the application of NA.

## Introduction

**FIGURE 1:**
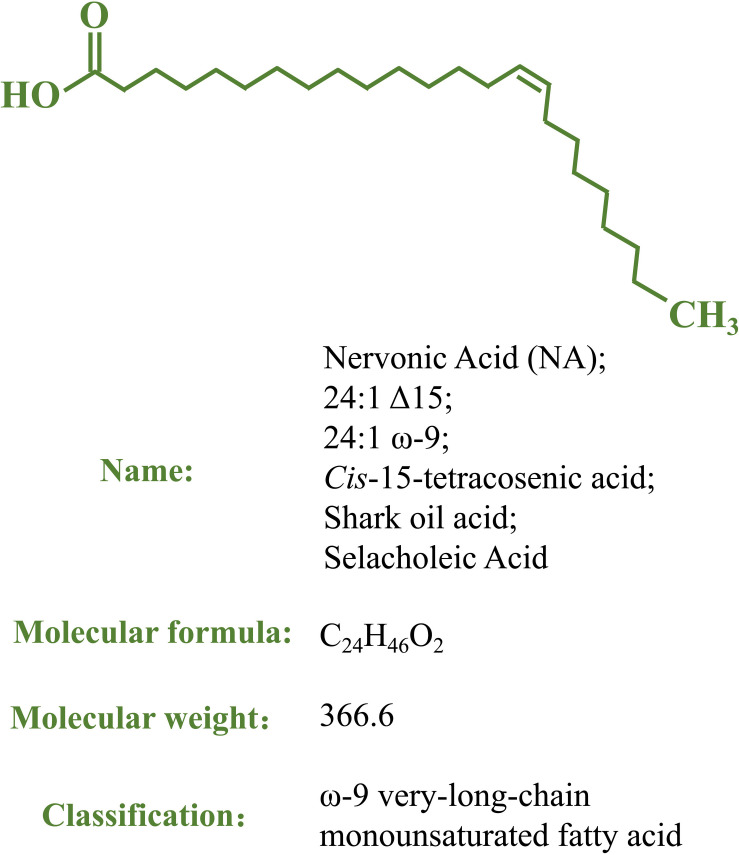
The structure and other basic information of nervonic acid.

Nervonic acid (NA; 24:1 Δ15, 24:1 ω-9; *cis*-tetracos-15-enoic acid) is a very-long-chain monounsaturated fatty acid (VLCMFA, 22–26 carbons) (Merrill et al., 1997; Figure 1) that was first isolated in shark brain and molecular structure was determined more than 100 years ago; it is also known as shark oil acid or selacholeic Acid (Tsujimoto and Kimura, 1926). It was found that the shark brain could repair itself in a short time after being severely damaged, suggesting the exceptional effect of NA in promoting the repair and regeneration of nerve fibers in damaged brain tissues (Sinclair and Crawford, 1972). NA combines with sphingosines via amide bonds to form nervonyl sphingolipids, which are chiefly found in nervous and brain tissues, comprising the white matter and myelin sheath of nerve fibers (Poulos, 1995; Merrill et al., 1997; Martínez and Mougan, 1998). NA plays a vital role in developing and maintaining the brain and biosynthesizing and improving nerve cells. NA is a natural component of maternal milk and can promote infant growth by assisting nervous system development (Farquharson et al., 1996; Ntoumani et al., 2013; Yu et al., 2019). Decreased NA levels are closely associated with a high risk of developing psychotic disorders in individuals (Coupland and Raoul, 2001; Amminger et al., 2012; Vozella et al., 2017), and supplementation with NA is an established effective treatment for symptoms of several neurological diseases, such as demyelinating disorders (Sargent et al., 1994; Vozella et al., 2017; Lewkowicz et al., 2019). NA can also function as a non-competitive inhibitor of human immunodeficiency virus type-1 reverse transcriptase (HIV-1 RT) in a dose-dependent manner (Kasai et al., 2002). Increasing dietary NA improves energy metabolism in mice and may be an effective strategy for the treatment of obesity and obesity-related complications (Keppley et al., 2020).

Over the past decades, a great number of sharks have been harvested in developed countries to obtain NA due to its market and commercial value. However, international organizations have banned shark fishing, resulting in shortages of NA resources. NA can be chemically synthesized with *cis*-13-docosenyl methyl ester as a precursor; however, the yield of NA by chemical synthesis is low and there are many by-products (Lei et al., 2013; Fan et al., 2018a). NA is also found in the seed oils of some wild plant species (Tang et al., 2013). However, long growth cycles, limited distributions, and highly variable seed yields have hindered the extraction of NA from natural plant sources (Tang et al., 2013). The construction of NA-producing microorganisms, microalgae, and plants via genetic engineering for nutraceutical and pharmacological applications offers a suitable approach that has attracted widespread attention due to its potential application in the large-scale production of NA (Guo et al., 2009; Taylor et al., 2009; Yuan et al., 2011; Umemoto et al., 2014; Huai et al., 2015; Fillet et al., 2017; Fan et al., 2018a; Xu et al., 2018; Liu et al., 2020). Recently, reviews on the genetic engineering of microorganisms and microalgae for the production of NA have been extensively conducted (Fan et al., 2018a; Li et al., 2019). Plant seed oil products are economical and sustainable industrial feedstock alternatives for shark fishing and traditional chemical synthesis (Leonard, 1994; Jadhav et al., 2005). To increase NA levels in seed oils, genetic engineering approaches have been used to improve existing plant sources and generate new elite cultivars, providing an alternative approach for creating novel cultivars with improved traits and desirable NA contents that cannot be obtained by traditional breeding methods (Giddings et al., 2000). Once a mature system is established in a plant, standard agricultural practices can produce significant amounts of target products, with the cultivation area being the only limitation. However, the use of transgenic plants to produce specific fatty acids (FAs) has not attracted much attention despite the market potential of these biological agents being very considerable. The existing information on the genetic engineering of plants for the production of NA is from isolated studies, and thus the relevant studies need to be thoroughly summarized and discussed to present an integrated and systematic evaluation.

In this review, the available information pertaining to NA biosynthesis, NA resources from plants, and the genetic engineering of NA biosynthesis in oil crops is summarized. Additionally, the factors affecting NA production in genetically engineered oil crops are discussed in detail, and the prospective trends in the engineering of NA are evaluated. Based on the NA biosynthesis pathway and assembly mechanism, new perspectives are highlighted and combined with existing reports on a genetic engineering strategy for the production of NA in plants. This review emphasizes the progress made toward various NA-related topics and explores the limitations and trends, thereby providing integrated and comprehensive insight into the nature of NA production mechanisms during genetic engineering. Furthermore, this report supports further work involving the manipulation of NA production through transgenic technologies and molecular breeding for the enhancement of crop nutritional quality or creation of plant biochemical factories to produce NA for use in nutraceutical, pharmaceutical, and chemical industries.

## NA Biosynthesis and Assembly

**FIGURE 2:**
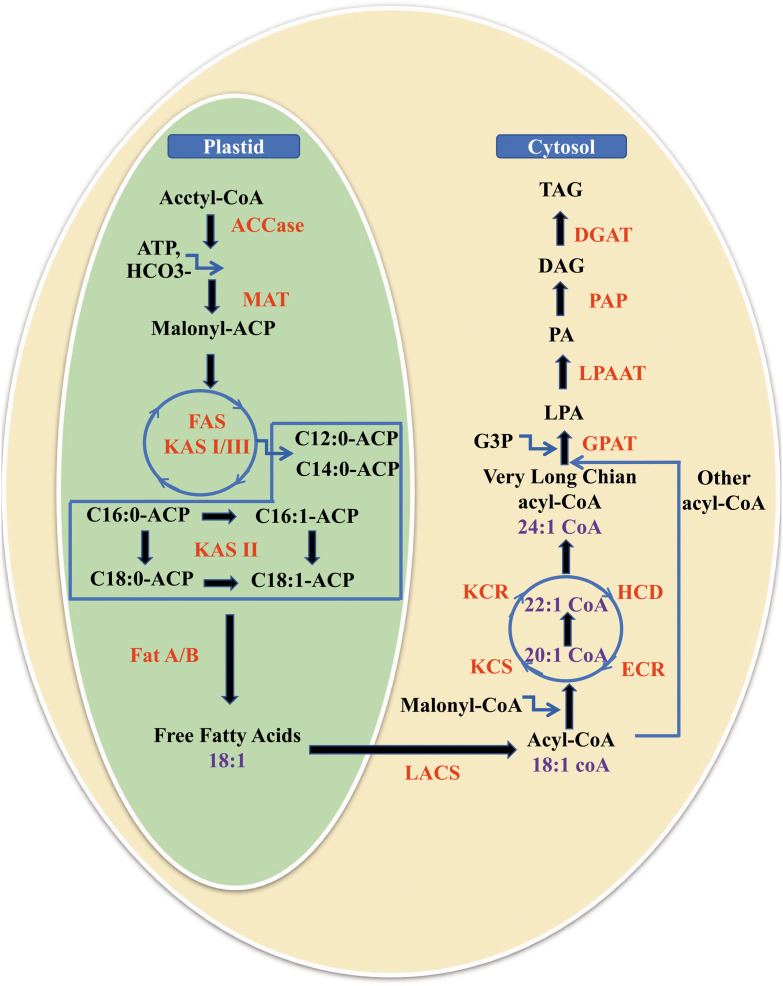
Biosynthesis and accumulation of nervonic acid in plants. It includes the *de novo* fatty acid synthesis in plant plastids, fatty acid elongation from oleic acid (18:1 ω-9) to nervonic acid (24:1 ω-9) for 3 cycles with each cycle adding two carbon units using four core enzymes located at the endoplasmic reticulum membrane in the cytoplasm, and fatty acids storage as triacylglycerols by three key acyltransferases. MAT, malonyl-CoA/ACP transacylase; FAS, fatty acid synthase; KAS, ketoacyl-ACP synthase; Fat A/B, fatty acid thioesterases A/B; LACS, long chain acyl CoA synthetase; FAE, fatty acid elongase; KCS, 3-ketoacyl-CoA synthase; KCR, 3-ketoacyl-CoA reductase; HCD, 3-hydroxacyl-CoA dehydratase; ECR, trans-2,3-enoyl-CoA reductase; GPAT, glycerol-phosphate acyltransferase; G3P, glycerol-3-phosphate; LPA, lysophosphatidic acid; LPAAT, lysophosphatidic acid acyltransferase; PA, phosphatidic acid; PAP, phosphatidic acid phosphatase; DAG, diacylglycerol; DGAT, diacylglycerol acyltransferase; TAG, triacylglycerol.

The biosynthetic pathway of NA includes *de novo* FA synthesis in plant plastids and fatty acid elongation (FAE) starting from oleic acid (18:1 ω-9) using four core enzymes located at the endoplasmic reticulum (ER) membrane in the cytoplasm (Figure 2) (Zakim and Herman, 1969; Baud and Lepiniec, 2009). Subsequently, NAs are assembled and stored in the form of triacylglycerols (TAGs) in organisms via the Kennedy pathway (Kennedy, 1961), in addition to transformation from phospholipids to TAGs (Bates et al., 2009) at the ER.

The *de novo* synthesis of FAs is catalyzed by FA synthases, which form enzyme complexes. It have been reported that FAs linked with an acyl carrier protein (ACP) are elongated to a chain length of C16 or C18 and are then released from fatty acyl-A by fatty acyl-ACP thioesterase (Fat A/B) (Figure 2) (Bonaventure et al., 2003; Wu and Xue, 2010; Tjellström et al., 2013). The released FAs are esterified rapidly by long-chain acyl-CoA synthetase (LACS) to prevent them from flowing out of the cells.

After the esterified FAs, including 18:1, are transported to the cytoplasm, FAs are converted into acyl-CoAs by LACS (Salas and Ohlrogge, 2002). Very long-chain fatty acids (VLCFAs, 22–26 carbons) including NA are synthesized in the form of acyl-CoAs by the FAE enzyme complex, which includes four core enzymes located on ER membranes (Haslam and Kunst, 2013). Each cycle of FAE adds two carbon units donated by malonyl-CoA to the acyl chain starting from 18:1 and involves four reactions. Firstly, malonyl-CoA and long-chain acyl-CoA are condensed by 3-ketoacyl-CoA synthase (KCS, sometimes designated as FA elongase, FAE), and secondly, the produced 3-oxoacyl-CoA is reduced by 3-ketoacyl-CoA reductase (KCR) yielding 3-hydroxyacyl-CoA. Then, the action of 3-hydroxacyl-CoA dehydratase (HCD) results in the generation of 2-enoyl-CoA. Finally, 2-enoyl-CoA is reduced by *trans*-2,3-enoyl-CoA reductase (ECR) to form elongated acyl-CoA (Figure 2) (Blacklock and Jaworski, 2002; Salas et al., 2005; Huai et al., 2015). In the end, 18:1 is elongated to 24:1 after three cycles of FAE.

These four enzymes have been characterized in *Arabidopsis thaliana* L. Heynh. (*A. thaliana*), which revealed that the last three enzymes (KCR, HCD, and ECR) function in all tissues exhibiting VLCFA biosynthesis and possess broad substrate specificity (Zheng et al., 2005; Paul et al., 2006; Bach et al., 2008; Joubès et al., 2008; Beaudoin et al., 2009). In contrast, KCS provides high substrate- and high tissue-specificities for FAE. Protein dynamic simulations revealed a discrepancy in the binding pockets between KCS proteins, which led to the hypothesis that the specificities of different products depend on the shape and size of the substrate-binding pockets (Haslam and Kunst, 2013). Therefore, the expression and activity of KCS determine the amount of synthesized product, and KCS is the rate-limiting enzyme of the VLCFA biosynthesis pathway (Joubès et al., 2008; Millar and Kunst, 2010; Chen et al., 2011; Huai et al., 2015; Yang et al., 2018). Moreover, it is evidenced that the final chain length of VLCFAs depends on the substrate specificity of KCSs (Kunst et al., 1992; Moon et al., 2001; Mietkiewska et al., 2004; Millar and Kunst, 2010; Haslam and Kunst, 2013). Over the past years, progress in understanding the biosynthesis of VLCFAs has been made by cloning and identifying *KCS* genes in different plants species (James et al., 1995; Lassner and Metz, 1996; Han et al., 2001; Das et al., 2002; Fofana et al., 2004; Mietkiewska et al., 2004, 2007a, b; Guo et al., 2009; Taylor et al., 2009; Huai et al., 2015; Wang et al., 2018; Yang et al., 2018; Li et al., 2020; Ma et al., 2020).

Fatty acids are mainly stored as TAGs, which are neutral lipids that are a major component of seed oil (Kaup and Thompson, 2002). TAG synthesis begins with the export of free FAs from the plastids followed by the stepwise acylation of these into the sn-1, sn-2, and sn-3 positions of glycerol backbone of glycerol-3-phosphate (G3P) (Kennedy, 1961; Lacey and Hills, 1996). The first acylation of G3P at the sn-1 position is catalyzed by glycerol-3-phosphate acyltransferase (GPAT) to form lysophosphatidic acid (LPA). The second acylation of LPA at the sn-2 position is catalyzed by lysophosphatidic acid acyltransferase (LPAAT) to form phosphatidic acid (PA). Dephosphorylation of the resultant PA is then catalyzed by phosphatidic acid phosphatase (PAP) to form DAG. The third acylation reaction, converting DAG to TAG, is catalyzed by diacylglycerol acyltransferase (DGAT) enzymes at the sn-3 position using a fatty acyl-CoA (Figure 2) (Kennedy and Weiss, 1956). The research findings showed that NA can only be incorporated into the sn-1 or sn-3 position instead of sn-2 position of TAGs (Scarth and Tang, 2006; Taylor et al., 2011; Umemoto et al., 2014; Fan et al., 2018b).

Previous studies suggested that MYB and bZIP transcription factors participated in regulating the synthesis of FAs (Yamamoto et al., 2009; Rong et al., 2020), and MYB played important roles in VLCFA biosynthesis particularly (Raffaele et al., 2008). A weighted gene co-expression network analysis in *Acer truncatum* Bunge (*A. truncatum*) showed that MYB and bZIP transcription factors were involved in regulating NA biosynthesis (Ma et al., 2020). Recent genome sequencing or transcriptome analysis of *A. truncatum*, *Malania oleifera* Chun et S. K. Lee (*M. oleifera*) or *Xanthoceras sorbifolia* (*X. sorbifolia*) species containing rich NA provides important foundations from which to study the molecular mechanisms influencing the NA production (Wang et al., 2018; Yang et al., 2018; Liang et al., 2019; Ma et al., 2019, 2020; Xu et al., 2019) and serves as a support point of NA production by genetic engineering.

## NA Resources From Plants

In the last 60 years, there has been much interest in obtaining new oil-containing plants for industrial and edible purpose (Jart, 1978). There has been particular focus on NA-containing oil seed plants to explore renewable resources and reduce shark fishing. Thus far, NA has been found in 38 plant species belonging to 31 genera and 13 families, of which eight woody and two herbaceous plants contain at least 4.6% NA (Ma et al., 2004; Fan et al., 2018a). The amounts of NA are affected by genetic factors and various environmental factors, such as planting pattern and growing conditions (Zheljazkov et al., 2012). Considering the great differences in the content of oil and nerve acid in the seeds of plants, only plants with high oil contents and abundant NA contents are suitable for exploiting and extracting NA (Zheljazkov et al., 2012). The seed oil content, NA and erucic acid (EA, 22:1) content, and NA production limitations of 10 valuable NA-containing plant species from seven families, including four woody and six herbaceous plants, have been listed and compared in Table 1.

**TABLE 1:** Summary of the information including seed oil content, NA and EA content, and NA production limitations on the main plant seeds rich in NA.

Species	Common name	Oil content (%)	NA content of total fatty acid (%)	EA content of total fatty acid (%)	Distribution	Problems	References
*Malania oleifera* Chun et S. K. Lee	/	58.0∼63.0	55.7∼67.0	∼13.1	Western Guangxi and southeast Yunnan, China	Rare, endangered, narrow distribution	Wang et al., 2006; Tang et al., 2013; Li et al., 2019; Xu et al., 2019
*Ximenia caffra* Sond.	Sour Plum	40.1∼65.0	5.9∼11.4	∼0	Tanzania, Zambia, Zimbabwe, Botswana, Namibia, Mozambique and South Africa	Lignoceric acid needs to be separated from NA	Lee, 1973; Venter and Venter, 1996; Chivandi et al., 2008
*Acer truncatum* Bunge	Purpleblow maple	45.0∼48.0	3.9∼7.8	∼17.0	North China, Japan, and Korea, North America and Europe	Eight to ten years to maturity	Appelqvist, 1976; Jart, 1978; Taylor et al., 2009; Qiao et al., 2018; Ma et al., 2019, 2020
*Xanthoceras sorbifolia* (*Xanthoceras sorbifolium* Bunge)	Yellowhorn	63.3∼69.5	2.0∼2.6	8.3∼8.5	North and Northeast China	Narrow distribution, a unique species in China	Zhang et al., 2017; Li et al., 2019; Liang et al., 2019
*Borago officinalis* L.	Borage	30.0∼40.0	∼1.5	1.5∼3.5%	Originated in Iran and some Mediterranean countries of West Asia, now in almost all over the world	Seeds shattered when mature	Appelqvist, 1976; Jart, 1978; El-Din and Hendawy, 2010; Hashemian et al., 2010
*Cannabis sativa* L.	Hemp	26.3∼37.5	3.0∼8.0	1.0∼14.0	Originated in India, Bhutan and Central Asia, now in various countries	Risk of drug harm seriously restricts the cultivation	Appelqvist, 1976; Jart, 1978; Kriese et al., 2004
*Cardamine graeca* L.	Bittercress	12.0∼13.0	45.0∼54.0	9.3∼10.0%	Mediterranean countries	Narrow red soil requirements, seed shattering	Jart, 1978; Taylor et al., 2009
*Lunaria annua/Lunaria biennis* L.	Honesty/money plant	25.0∼35.0	14.0∼24.2	43.0∼50.0	Europe to West Asia	A poor-yielding biennial, seed shattering	Mastebroek and Marvin, 2000; Taylor et al., 2009; Dodos et al., 2015
*Tropaeolum speciosum* Poepp. & Endl.	Flame Flower	12.3∼26.0	40.0∼45.4	∼17.0	Rare plant growing in Chile	Difficult to obtain seeds and reproduce	Litchfield, 1970; Taylor et al., 2009; Li et al., 2019
*Tropaeolum majus* L.	Nasturtium	6.0∼10.0	1.0∼2.0	75.0∼80.0	Originated in South America Peru, Brazil and China, now in temperate areas over the world	Low oil content, difficult to obtain seeds and reproduce	Taylor et al., 2009

*NA, nervonic acid; EA, erucic acid.*

Plants with an oil content of more than 10% have potential industrial value. As indicated in Table 1, nine out of the 10 plant species have oil contents greater than 10%. The high oil content (≥40.1%) is found in the seeds of *X. sorbifolia*, *Ximenia caffra* Sond., *M. oleifera*, and *A. truncatum.* Given a certain oil content, the ratio of NA to total FAs can best reflect the utilization value of a plant species as a raw material for NA extraction. Eight out of 10 of the plant species have NA contents greater than 2%. The highest NA content (≥63.0%) is found in the seeds of *M. oleifera*, *Cardamine graeca* L., and *Tropaeolum speciosum* Poepp. & Endl (*T. speciosum*). Considering the above two factors, among the 10 plant species, the seeds of *M. oleifera*, *T. speciosum*, and *Lunaria annua* L./*Lunaria biennis* Moench (*L. annua*) have both high oil and NA contents and are thus potential candidates for the development of NA products.

Nervonic acid is the major FA in *M. oleifera* and accounts for 55.7–67.0% of the total FAs in this plant, the oil content of which ranges from 58.0% to 63.0%. *M. oleifera* has the highest content of NA reported thus far in any seed fat. However, *M. oleifera* is a rare and endangered woody oil plant that grows naturally in western Guangxi and southeast Yunnan in China, and its narrow distribution in addition to its protected status limits its development and industrialization (Wang et al., 2006; Tang et al., 2013; Li et al., 2019; Xu et al., 2019). *T. speciosum*, a unique perennial herbaceous plant species from Chile, has a seed oil content in the range of 12.3∼26.0%, with NA remarkably constituting 40.0∼45.4% of the total FA content. However, its seeds are difficult to obtain and reproduce (Litchfield, 1970; Taylor et al., 2009; Li et al., 2019). *L. annua* is a biennial herbaceous oil crop that is considered as a niche crop beyond its ornamental value in many countries. The oil content of its seeds is 25%∼35%, with NA accounting for 14.0∼24.2% of the total FA content but with EA constituting up to 50%. The seed yields of *L. annua* vary greatly (800–2000 kg/ha), and seed shattering and the associated harvesting difficulties are long-standing problems (Mastebroek and Marvin, 2000; Taylor et al., 2009; Dodos et al., 2015). In addition, due to its high EA content, *L. annua* oil cannot meet the nutraceutical and pharmaceutical NA oil requirements of a high NA content but very low EA (< 5%) content, as diets rich in EA have toxic effects on the heart (Bettger, 2000; Das et al., 2002; Taylor et al., 2009).

There are three key and complex issues for NA production from oil seeds. First, some species suffer from highly variable seed yields, and seeds are prone to shattering when matured and may fall to the ground in the process of harvesting. Second, some species are only distributed in specific areas, which restricts their wide application. Third, some seed oils require the removal of erucic or lignoceric acid by distillation or other expensive methods before they can be used in health applications. Therefore, although a number of species present excellent oil or NA contents, only *L. annua* and *A. truncatum* are considered niche species in practice (Ma et al., 2019, 2020), and the other plant species must be further researched to address the current NA extraction inefficiencies. Genetic modification may thus be an excellent approach for the mass production of NA.

## Genetic Engineering of NA Biosynthesis in Oil Crops

Nervonic acid is not present in the main cultivated vegetable oil crops (e.g., soybean, rapeseed, sunflower), and the low proportion of NA in natural oilseeds limits its production (Taylor et al., 2009; Li et al., 2019). Breeding programs have been intermittently ongoing in Europe to develop annual types of *L. annua* aiming to achieve increased oilseed production thus higher NA production, but the results have been less than optimal (Mastebroek and Marvin, 2000). The genetic engineering of oil crops for NA production has thus drawn increasing interest. Genetic engineering provides an alternative method for developing novel varieties with improved traits and desirable characteristics that cannot be achieved by traditional breeding approaches (Giddings et al., 2000). Many studies have shown that the expression of a recombinant KCS in plant hosts alters the seed oil VLCFA contents (Ghanevati and Jaworski, 2001; Das et al., 2002; Mietkiewska et al., 2004, 2007a, b, 2008; Kanrar et al., 2006), and thus the use of *KCS* or other genes from the NA biosynthesis pathway for increasing NA contents in seed oils via genetic engineering offers a potential effective strategy.

There have been limited attempts to improve NA contents via genetic engineering in oil crops thus far, though some remarkable success has been achieved through the heterologous expression of KCS in transgenic oilseeds. A general comparison of the various transgenic seeds of NA oils is provided in Table 2. Guo et al. (2009) identified the *KCS* gene from *L. annua* (LaKCS). The expression of *LaKCS* in yeast led to the biosynthesis of NA in transgenic yeast, which is not normally present in wild-type yeast cells. The expression of LaKCS in *A. thaliana* and a high-EA *Brassica* crop *Brassica carinata* (*B. carinata*) under the control of the seed-specific promoter napin led to a 30–40-fold increase in the NA content in transgenic *A. thaliana* and 7–10-fold increase in the NA content in transgenic *B. carinata* in comparison to the wild-type. The highest NA content in transgenic *B. carinata* offspring was 30%, whereas it was 2.8% in the wild plant and 20% in *L. annua*; however, the EA content in this best transgenic *B. carinata* line was 25%, whereas it was 35.8% in the wild plant and 44% in *L. annua* (Guo et al., 2009).

**TABLE 2:** Comparison of the research on improving the NA content by means of genetic engineering in oil crops.

Gene	Donor species	NA/EA content of donor species (%)	Receptor species	NA/EA content of control (%)	Strategy	NA/EA content of transgenic receptor species (%)	Fold change	Substrate in WT and KCS activity	References
*LaKCS*	*Lunaria annua*	20/44	Yeast *(Saccharomyces cerevisiae)*	0/0	*GAL1:LaKCS*	Can catalyze NA/EA synthesis, EA > NA	Increase	Low EA substrate in WT, KCS activity: 20∼30-fold Higher	Guo et al., 2009
			*Arabidopsis thaliana*	0.19 ± 0.01/2.0 ± 0.2	*Napin:LaKCS*	4.3 ± 0.5 (the best offspring line 7.6)/13.8 ± 0.8	30∼40-fold increase/sevenfold increase	Low EA substrate in WT	
			*Brassica carinata*	2.8 ± 0.1/35.8 ± 1.9		19.5 ± 3.8 (the best offspring line 30)/ 29.0 ± 2.6	7∼10-fold increase/1.2-fold decrease	High EA substrate in WT	
*CgKCS*	*Cardamine graeca*	46/10	Yeast *(Saccharomyces cerevisiae)*	0/0	*GAL1:CgKCS*	Can catalyze NA/EA synthesis, EA≈NA	Increase	Low EA substrate in WT, KCS activity: 10∼42-fold Higher	Taylor et al., 2009
			*Arabidopsis thaliana*	0.2/2	*Napin:CgKCS*	7∼11/6∼8	35∼55-fold increase/3∼4-fold increase	Low EA substrate in WT	
			*Brassica carinata*	2.9 ± 0.1/38.5 ± 0.4		42.2 ± 1.3 (the best offspring line 44)/ 5.6 ± 0.8	15-fold increase/6.9-fold decrease	High EA substrate in WT	
			*Brassica napus*	1.1 ± 0.0/56.1 ± 0.0		31.3 ± 5.0/21.6 ± 4.0	28-fold increase/2.6-fold decrease	High EA substrate in WT	
*LaKCS*	*Lunaria annua*	20/44	*Camelina sativa*	0/3.4	*Gly-P:LaKCS*	5.9∼12.0/10.5∼11.6	Increase/3.3-fold increase	Low EA substrate in WT	Huai et al., 2015
*LaKCS* and *AtKCR*	*Lunaria annua* and *Arabidopsis thaliana*	20/44 and 0.2/2			*Gly-P:LaKCS* + *Gly-P:AtKCR*	11.3∼11.8/11.2∼11.5	Increase/3.3-fold increase	Low EA substrate in WT	
*LaKCS* and *AtHCD*	*Lunaria annua* and *Arabidopsis thaliana*	20/44 and 0.2/2			*Gly-P:LaKCS* + *Gly-P:AtHCD*	10.7∼12.9/11.2∼11.5	Increase/3.3-fold increase	Low EA substrate in WT	
*LaKCS* and *AtECR*	*Lunaria annua* and *Arabidopsis thaliana*	20/44 and 0.2/2			*Gly-P:LaKCS* + *Gly-P:AtECR*	9.9∼10.0/11.9∼12.5	Increase/3.6-fold increase	Low EA substrate in WT	
*LaKCS*, *AtKCR* and *AtHCD*	*Lunaria annua* and *Arabidopsis thaliana*	20/44 and 0.2/2			*Gly-P:LaKCS* + *Dleo-P:AtKCR* + *Gly-P:AtHCD*	10.0∼10.8/11.4∼12.1	Increase/3.5-fold increase	Low EA substrate in WT	
*MoKCS11*	*Malania oleifera*	40/15	*Arabidopsis thaliana*	0.2/2.4	*Phaseolin:MoKCS11*	5.0/8.0	25-fold increase/3.3-fold increase	Low EA substrate in WT	Li et al., 2020
			*Brassica napus*	1.2/24.4		1.2/31.2.	no increase/1.3-fold increase	High EA substrate in WT	
			*Camelina sativa*	0.5/3.7		2.5/5.1	5-fold increase/1.4-fold increase	Low EA substrate in WT	

*NA, nervonic acid; EA, erucic acid; GAL1, galactose-inducible GAL1 promoter; Napin, seed-specific napin promoter; Gly-P, seed-specific soybean glycinin-1 promoter; Dleo-P, strong seed specific soybean oleosin promoter; WT, wild-type.*

The *KCS* gene isolated from *C. graeca* (CgKCS) was transferred in yeast, which resulted in the biosynthesis of NA in transgenic yeast (Taylor et al., 2009). The expression of CgKCS in *A. thaliana*, *B. carinata*, and high-EA *Brassica napus* (*B. napus*) under the control of the seed-specific promoter napin resulted in a 35–55-fold increase in NA content in transgenic *A. thaliana*, a 15-fold increase in NA content in transgenic *B. carinata*, and a 28-fold increase in transgenic *B. napus* in comparison to the wild-type. The maximum NA content in the *B. carinata* transformant reached 44%, whereas it was 2.9% in the wild plant and 46% in *C. graeca*; however, the EA content in the *B. carinata* transformant was only 5.6%, whereas it was 56.1% in the wild plant and 10% in *C. graeca* (Taylor et al., 2009).

The *MoKCS11* gene isolated from *M. oleifera* was expressed in *A. thaliana*, high-EA *B. napus* and *Camelina sativa* (L.) Crantz. (*C. sativa*) under the control of the seed-specific promoter phaseolin resulted in 5% NA accumulation in transgenic *A. thaliana*, no expected increase in NA content in transgenic high-EA *B. napus*, and 2.5% NA accumulation in transgenic *C. sativa* (Li et al., 2020). *MoKCS11* may not synthesize NA in *B. napus*, even though it has abundant EA as the direct substrate of NA. Overexpression of *MoKCS11* results in similar level of NA accumulation in *A. thaliana and C. sativa* probably because there is a similar and rich proportion of 20:1 in their seeds and *MoKCS11* may have a substrate preference for 20:1 instead of EA *in planta* (Li et al., 2020).

The above research attempted to overexpress KCS to increase the NA content. To understand the impact of KCR, HCD, and ECR in the FAE enzyme complex on NA production, Huai et al. (2015) systematically evaluated the combinatorial effects of KCR, HCD, and ECR with KCS on NA production. LaKCS and AtKCR, and AtHCD and AtECR from *A. thaliana* were used for the assessment. Five single gene or gene combinations, including *LaKCS* alone, *LaKCS* with *AtKCR*, *LaKCS* with *AtHCD*, *LaKCS* with *AtECR*, and *LaKCS* with *AtKCR* and *AtHCD*, driven by the seed-specific soybean glycinin-1 or oleosin promoter, were overexpressed in *C. sativa*. The NA content in the transgenic *C. sativa* seed oil increased from null to 6–12% in the LaKCS transgenic lines. However, the NA content did not increase further when one or two additional genes in the FAE enzyme complex were expressed in the mature seeds. At the early stage of seed development, the NA contents from the LaKCS, AtKCR, and AtHCD co-expressing lines were significantly higher than that of the LaKCS transgenic lines, while the ultimate NA content did not change significantly. The maximum NA content of 12.9% was attained in the *C. sativa* LaKCS and AtHCD co-expressed line, whereas it was 0% in the wild plant, 20% in *L. annua*, and 0.2% in *A. thaliana.* However, the EA content in this best transgenic line was 11.5%, whereas it was 3.4% in the wild plant, 44% in *L. annua*, and 2% in *A. thaliana* (Huai et al., 2015).

## Factors Affecting NA Production in Genetically Engineered Oil Crops

An understanding of the regulatory features controlling NA biosynthesis is critical for the future engineering of oilseed crops for increased NA production. Several factors influence the application of transgenic techniques to promote the efficient improvement of NA in oil-producing plants.

### KCS Is Decisive Instead of KCR, HCD, and ECR

Over the past few decades, it has been demonstrated that KCS is a rate-limiting enzyme and regulating the expression of specific KCS influences the final chain length and contents of VLCFAs (Cahoon, 2000; Han et al., 2001; Mietkiewska et al., 2004, 2007a, b; Haslam and Kunst, 2013). Huai et al. (2015) further confirmed this conclusion specifically regarding NA synthesis, in that the final amount of NA product is determined by the amount of KCS rather than that of KCR, HCD, and ECR (Table 2).

### Appropriate KCS Enzymes Need to Be Used

Nervonic acid is synthesized at the expense of EA and 20:1 FA. *KCS* genes from different sources exhibit distinct substrate specificity and thus possess a differential ability and efficiency to convert substrates to NA, even in the same receptor species. *B. carinata* transformed with *LaKCS* produced oil with 30% NA, but the EA content was still 29.0%. When transformed with the *CgKCS*, *B. carinata* produced oil with up to 44% NA and a significantly reduced EA level of 5.6% (Table 2). Since EA is the predominant VLCFA in wild-type *B. carinata*, this suggests that CgKCS performed better than LaKCS in transgenic *B. carinata* to convert EA to NA.

Similarly, *A. thaliana* transformed with *LaKCS* produced oil with 7.6% NA and 13.8% EA, while transformation with *CgKCS* produced oil with 7–11% NA and 6–8% EA (Guo et al., 2009; Taylor et al., 2009; Table 2). In *A. thaliana LaKCS* transgenics where 20:1 substrate is the predominant VLCFA in the wild-type, the proportion of EA is about threefold higher than that of NA (Guo et al., 2009). This indicated that these *KCS* genes have a strong capacity to convert 20:1 to EA instead of EA to NA. Notably, in *A. thaliana CgKCS* transgenics, CgKCS has the ability to convert 20:1 into EA and to produce NA using EA as the precursor, so that NA is as prominent a product (7–11%) as EA (6–8%) in the transgenic oilseed (Taylor et al., 2009; Table 2). This indicates that CgKCS exhibits a strong capacity to convert 20:1 into EA as well as to convert EA into NA. For another example, the overexpression of *KCS* genes from *B. napus* (Han et al., 2001), *Tropaeolum majus* L. (Mietkiewska et al., 2004), *Brassica. juncea* (Kanrar et al., 2006), *Crambe abyssinica* (*C. abyssinica*) (Mietkiewska et al., 2007a), and *M. oleifera* (Li et al., 2020) in high-EA Brassicaceae results in significantly improved EA, but not NA proportions, as these *KCS* genes elongate 20:1-CoA more effectively. In addition, the *Teesdalia nudicaulis* (L.) W. T. Aiton KCS preferentially elongates 18:1 substrates in yeast and *A. thaliana* (Mietkiewska et al., 2007b). Thus, the substrate selectivity of the KCS enzyme is indeed relatively promiscuous, and similar results have been observed in experiments where LaKCS and CgKCS are ectopically expressed in yeast, which indicated rather loose specificity via the detection of each of three labeled substrates 18:1, 20:1, and 22:1 (Guo et al., 2009; Taylor et al., 2009).

The evolution of *KCS* genes has resulted in their functional divergence and renovation, and thus a wide range of substrate specificities and product diversities exist (Fan et al., 2018a). Due to the membrane-binding properties of the KCS protein, our understanding of the nature and regulation of this enzyme is still limited, and the substrate specificity of KCS needs be investigated (Puyaubert et al., 2005a, b). In present studies, *KCS* genes have been selected chiefly from donor species that have high NA levels and have been considered as niche crops but with limited success because of uneconomy. For instance, *C. graeca* has narrow red soil requirements, a low oil content, and seeds that are prone to shattering (Jart, 1978; Taylor et al., 2009), and *L. annua* is a poor-yielding biennial with a low oil content and shatter-prone seeds (Mastebroek and Marvin, 2000; Guo et al., 2009; Dodos et al., 2015; Huai et al., 2015). In the future, numerous KCS proteins for VLCFA biosynthesis need to be identified in plant resources with high NA contents, and their functions, especially the mechanisms by which KCS affects NA production, need to be verified. Since NA is particularly abundant in *M. oleifera*, *T. speciosum*, and *A. truncatum*, new KCS enzymes discovered and identified from these plants may constitute good choices in NA genetic engineering (Ma et al., 2004, 2019, 2020; Xu et al., 2019).

### Seed-Specific Promoters, High Transgene Copy Numbers and/or the Optimum Genomic Integration Sites, and High KCS Activity All Contribute to Increased Proportions of NA

The overexpression of *KCS* genes with a strong seed-specific promoter in genetically engineered oilseed plants is a key avenue for improving NA production by increasing the expression of KCS in the seeds specifically and decreasing the potential risk of the constitutive expression of *KCS* genes. The seed-specific napin promoter (Guo et al., 2009; Taylor et al., 2009), the seed-specific soybean glycinin-1 promoter (Huai et al., 2015), and the strong seed-specific soybean oleosin promoter (Huai et al., 2015) were used to drive KCS expression in *A. thaliana* (Guo et al., 2009; Taylor et al., 2009), *B. carinata* (Guo et al., 2009; Taylor et al., 2009), *B. napus* (Taylor et al., 2009) or *C. sativa* (Huai et al., 2015), leading to 7–55-fold increases in NA content (Table 2).

The transcription levels of different genes under the control of the same promoter are quite different and the same gene may exhibit a similar transcription level under the regulation of different seed-specific promoters (Huai et al., 2015). That is to say, the level of gene transcription is also limited by the gene property, even sometimes by the promoter as we have known. The promoter affects the gene expression level, but the maximum expression level is determined by the gene. The stability and decay rate of individual mRNA molecules are affected by gene sequence GC%, DNA sequence elements, or the secondary structure of mRNAs, and so some genes are high-expression genes and others are not.

The increased NA phenotype of *LaKCS* transgenic lines was found to be associated with multiple *LaKCS* transgene copies, which led to higher transcript intensities, though the correlation was not necessarily linear (Guo et al., 2009). Similarly, research on ACP thioesterase transgenes at the level of the major target FA confirmed that in order to achieve the maximum expression levels of target FA, it is important to select high copy numbers and/or the optimal genomic integration sites for improving transgenic oil quality (Tang et al., 2003).

Upon providing 14C-22:1-CoA as a substrate, the KCS activity from the developing seeds of transgenic *LaKCS-B. carinata* and *CgKCS-B. carinata* was 20–30-fold and 10–42-fold higher, respectively, than the barely detectable erucoyl-CoA elongation activity exhibited by the wild-type control plants (Guo et al., 2009; Taylor et al., 2009; Table 2). Furthermore, the 22:1-CoA KCS activity levels in the developing *B. carinata* seeds were well-correlated with the associated *LaKCS* or *CgKCS* transcript signals according to northern blot analysis (Guo et al., 2009; Taylor et al., 2009).

### Sufficient Specific Substrate Is Available in Receptor Species

In addition to KCS specificity and the amount of KCS expression, the availability and magnitude of specific substrates of KCS enzymes is also a key factor influencing the efficiency of chain elongation and ultimately the proportion of the NA produced by genetic engineering (Guo et al., 2009; Taylor et al., 2009; Huai et al., 2015; Yu et al., 2017).

Considering that EA is the major precursor of NA, if a high-EA crop contains only little NA in its seed oil due to the absence of KCS with a strong capacity to convert EA to NA, then this crop is a good option as an acceptor species for NA genetic engineering via the transformation of an appropriate *KCS* gene. This is supported by studies where the *KCS* genes from *L. annua* (Guo et al., 2009; Huai et al., 2015) and *C. graeca* (Taylor et al., 2009) have been overexpressed in *B. carinata* (Guo et al., 2009; Taylor et al., 2009) and *B. napus* (Taylor et al., 2009) with high EA, resulting in a significant improvement in NA proportion.

Heterologous expression of the *LaKCS* gene in *A. thaliana, B. carinata*, and *C. sativa* increased the NA content to 4.3–7.5%, 19.5–30%, and 5.9–12.0%, respectively (Guo et al., 2009; Huai et al., 2015; Table 2). A similar phenomenon has also been observed in other studies. Heterologous expression of the *CgKCS* gene in *A. thaliana*, *B. carinata*, and high-EA *B. napus* increased the NA content to 7–11, 42.2, and 31.3%, respectively (Taylor et al., 2009; Table 2). The NA quantities in the seed oil from engineered *A. thaliana* and *C. sativa* were low, and these plants have lower EA contents of 2 and 3.4%, respectively, in their wild-type seeds (Guo et al., 2009; Taylor et al., 2009; Huai et al., 2015; Table 2). In contrast, the NA contents in seed oil of engineered *B. carinata* and high-EA *B. napus* were significantly enhanced, and these plants have much higher EA levels of 35.8 and 56.1% for elongation in their wild-type seeds (Guo et al., 2009; Taylor et al., 2009; Table 2). Therefore, the proportion of EA substrate in the oilseed may limit the degree of NA synthesis. This indicates that not only KCS specificity, but also substrate availability, exerts a certain control on NA production.

The availability of sufficient specific substrates such as EA may exist in natural receptor species or be created via transgenes. It was reported that EA can accumulate in *C. abyssinica* by reducing the desaturase activity by co-expressing the LdLPAAT, BnFAE1, and CaFAD2-RNAi genes. Therefore, more 18:1 can enter the FA extension pathway, resulting in a 28.0% higher EA level than that in the wild-type (Li et al., 2012). This strategy of creating receptors with high EA substrates can be used to encourage NA accumulation in genetic engineering.

### Other Optimal Participants in VLCFA Elongation Are Available in Receptor Species

It is not necessarily the case that a high abundance of EA leads to higher NA production. The NA content in *CgKCS* transgenic *B. carinata*, with a lower EA substrate level of 35.8% in the wild-type seeds, reached 42.2%, while an NA content of 31.3% was achieved in *CgKCS* transgenic *B. napus*, which has a high EA level of 56.1% (Taylor et al., 2009; Table 2). The difference appears to be more complex than simply KCS substrate availability. Indeed, the higher level of EA in *B. napus* could not necessarily be translated into NA efficiently, which may be a result of four possibilities. First, even if there is a strong napin-driven overexpression of *CgKCS*, the KCS of native plants still has a considerable contribution to FA products, which reflects a varying number of elongation cycles starting from various indigenous 18:1 or VLC primers. Second, the lower conversion of EA to NA in high-EA *B. napus* is possibly limited by the activities of the other three enzymes of the elongase system. Third, NA synthesis in high-EA *B. napus* may lack other rate-limiting elongation substrates such as malonyl-CoA, in which case it is required to co-express a cytosolic acetyl-CoA carboxylase to facilitate elongation. Fourth, there may also be inadequate reductants (NADH and/or NADPH) for the second or fourth enzymatic steps in the elongation complex. The variable effects of the reliance of *KCS* gene expression on the receptor plant have significant implications for the selection of suitable receptor platforms.

### There Is Efficient Assembly of NA to More TAG Sites

Compared with *B. carinata*, the considerably less than complete conversion of EA to NA *in B. napus* (Taylor et al., 2009; Table 2) may be due to the disadvantages in the selectivities or preferences of acyltransferases with regard to the degree of assembling NA to sn-1 and/or sn-3 sites of TAGs; therefore, the substrate specificity of the three acyltransferases can be modified to accumulate more NA in the cells.

Nervonic acid can only be incorporated into the sn-1 or sn-3 position of TAGs through endogenous acyltransferases, but not into both positions at the same time, and is predicted more likely to be incorporated into sn-3. However, C16 and C18 FA are typically found instead of NA in the sn-2 position in the fungus *Mortierella capitata* RD000969, microalga *Mychonastes afer* (Umemoto et al., 2014; Fan et al., 2018b). Similar limitation of further increase NA content also occurs in high-EA *B. napus* and *B. carinata* and negligible NA is found at the sn-2 position on the glycerol backbone because the ability of endogenous *Brassica* LPAAT to incorporate nervonoyl moieties into sn-2 position is very low (Scarth and Tang, 2006; Taylor et al., 2011). This bottleneck also limits the content of EA and other VLCFAs as the longer FAs tend to be localized at the sn-1,3 position, which restricts the level of NA, EA or other VLCFAs in the seed oil to a theoretical limit of 66%. One successful study resulted in increases of EA of up to 56% compared to 45% in the control via the expression of the yeast SLC1-1 in high-EA *B. napus* (yeast LPAAT, Zou et al., 1997). However, expressing *Limnanthes* LPAATs in high-EA *B. napus* only led to an increased proportion of erucoyl moieties at the sn-2 position of up to 41%, instead of enhancing the total EA content. Given these EA engineering results, it is inferred that NA content is limited by various factors, and the improved ability of LPAAT to incorporate nervonoyl moieties into sn-2 could raise the upper limit but not necessarily enhance the end NA content. In the future, it is more feasible to modificate the substrate specificity of GPAT and LPAAT and co-expressing multiple genes in *B. carinata* or high-EA *B. napus* to increase NA levels over 66% in seed oil (Cahoon et al., 2007; Taylor et al., 2011).

## Cruciferae Oil Crops With Favorable Attributes Are Ideal Receptor Species

Regarding an ideal receptor platform, there has been special focus on Cruciferae. Many cruciferous seeds are high in oil, and numerous cruciferous oils contain ample VLCFAs, affording a superior basis for the synthesis of high amounts of NA in the seed oil. Contrary to most other oil crops, this family exhibits good growth capability in temperate climates and high harvest yields. Additionally, the transformation efficiency of some cruciferous species is particularly high. Thus, cruciferous oilseed crops with favorable attributes are becoming increasingly popular as industrial platforms for engineering NA oil production.

For example, *B. carinata* is an attractive receptor species that was used for the expression of LaKCS and CgKCS (Guo et al., 2009; Taylor et al., 2009). *B. carinata* is resistant to fungal diseases, drought, weeds, and insect pests and is thus suitable for growth in a wide range of regions. It also has a very high yield of 2500–3000 kg/ha (Taylor et al., 2009) and is very easily transformed (at a rate of 25–50%, Babic et al., 1998). However, high-EA *B. napus*, which was previously used to express CgKCS for engineering NA oil production, has a yield of only 1500 kg/ha. In addition, the transformation efficiency of high-EA *B. napus* is typically poorer than that of canola cultivars (Taylor et al., 2009). *C. sativa* is becoming a popular industrial platform for the production of FA oil by means of genetic engineering (Lu et al., 2011; Nguyen et al., 2013; Cahoon, 2014; Carlsson et al., 2014; Iskandarov et al., 2014; Nguyen et al., 2015) and was used to express LaKCS for NA oil production (Huai et al., 2015). This species has the advantages of cold and drought tolerance and low fertilizer requirements and is also distributed widely. Further, it has a short lifecycle (100–120 days) and is amenable to transformation using *Agrobacterium*-mediated methods (Gugel and Falk, 2006; Pilgeram et al., 2007; Lu and Kang, 2008).

The average oil content of the mature seeds of *C. graeca* is about 13%, and the oil content of *B. carinata* is 38%, which is about 10% lower than in high-EA *B. napus* varieties (Taylor et al., 2008). The expression of CgKCS had no adverse effect on oil content (Taylor et al., 2009). The average percentage of NA found in transgenic *B. carinata* is 42.2%, which is similar to the value of 46% in *C. graeca*, but the oil content of *B. carinata* is three times that of *C. graeca* and the yield of *B. carinata* is about 2500–3000 kg/ha (Taylor et al., 2009; Table 2). The projected NA content, seed oil content, and high field seed yields make *CgKCS* transgenic *B. carinata* lines the best performers among all NA seed sources thus far. However, in order to produce ultra-high NA in this crucifer as an acceptable new platform crop, the seed oil content of *B. carinata* needs to be significantly improved, which could be achieved through genetic modification, such as overexpressing the Arabidopsis *DGAT1* gene (Jako et al., 2001) or the yeast *SLC1-1* gene (Zou et al., 1997; Katavic et al., 2001; Taylor et al., 2001).

## A Superior Growth Environment and Manual Selection Contribute to NA Production

The profiles of the *CgKCS* transgenic *B. carinata* lines are from confined field trials (Taylor et al., 2009), while the *LaKCS* transgenic *B. carinata* lines translate well from greenhouse to field conditions (Katavic et al., 2001; Taylor et al., 2001). In terms of industrial-scale engineering of NA oil production in oil crops, climate, geographical location, and hydrology are all important factors that should be considered, as these factors affect the oil content, yield, and the FA profile, ultimately significantly influencing NA production. Appropriate temperatures, sufficient sunshine, and a good supply of water and fertilizer are generally conducive to both the filling of oil seeds as well as oil accumulation. However, temperature extremes and low light, drought, and flooding will greatly reduce the oil content.

Following genetic analysis and gene expression, the best transgenic lines with a high-NA phenotype are manually selected according to the FA profile in each generation, which is conducive to high NA production (Guo et al., 2009; Taylor et al., 2009; Huai et al., 2015). The NA content in transgenic Arabidopsis T_2_ seed oils expressing the LaKCS was 4.3 ± 0.5%, and after selection the best line T_3_ transgenic line had an NA content of 7.6%. The NA content in transgenic *B. carinata* T1 seed oils expressing LaKCS was 19.5 ± 3.8%, and following selection, the best NA content in the T2 transgenic line was 30% (Guo et al., 2009). On average, the NA content in the *CgKCS* transgenic *B. carinata* T_1_ seed oils was 38%, and after selection, the highest NA level in the T_4_ transgenic line reached 44% (Taylor et al., 2009; Table 2).

## Conclusion and Perspectives

The brain and nervous system are composed of white matter and gray matter. Docosahexaenoic acid (DHA, 22:6) are typical FAs especially concentrated in the gray matter, and NAs are typical FAs of the white matter (Sinclair and Crawford, 1972; Poulos, 1995; Merrill et al., 1997; Martínez and Mougan, 1998; Brenna and Diau, 2007; Weiser et al., 2016). Like DHA, arachidonic acid (eicosatetraenoic acid, ARA, 20:4), eicosapentaenoic acid (EPA, 20:5), and conjugated linoleic acid, NA is a strong candidate for further evaluation as a lipid supplement with brilliant application prospects due to its excellent biofunctions in promoting human health (Coupland and Langley, 1991; Coupland, 1996; Coupland and Raoul, 2001). Higher levels of NA in the diet assist in the formation of the myelin membrane outside the myelinated nerve fibers and also bind to transcription factors and regulate gene expression, thus modulating lipid and energy metabolism (Zheng et al., 2006; Van Meer et al., 2008). While an increasing number of studies have focused on the biological functions of NA, our current understanding of NA is still limited in that systematic and reliable studies on the role of NA in the general population are still lacking and the mechanisms of how dietary and physiological factors affect NA levels and molecular forms in animals and humans need to be specified. Natural or engineered seed oils with enriched NA but very low EA content (<5%) could be used as the necessary feedstocks to study health disease models through feeding trials on humans and animals in nutraceutical and pharmaceutical applications (Bettger, 2000). Achieving a balance between NA and EA is thus an important goal. Future NA products aim to develop edible NA oils^1^ and produce dairy products enriched in NA by adding high-NA feed to dairy livestock for human consumption (Bettger, 2000; Bettger et al., 2003), thus providing a healthy alternative to oil or milk powder for enhancing neural development in infants and preventing neurodegenerative diseases.

The use of NA in chemical applications is promising. Lunaria oil containing 14.0–24.2% NA can be used as an industrial lubricant (Meier zu Beerentrup and Röbbelen, 1987; Soest, 1994). NA has high potential for the synthesis of nylon 15, provided that there is a low EA in the oil mixture, and NA can be processed into pentadecane diacid for the production of polyesters (Jart, 1978). Nervonoyl-CoA has been identified to be able to produce new wax esters in transgenic *Brassica* crops as a desired precursor/intermediate (Taylor et al., 2009).

Given these research and application requirements, future research should focus on industrial NA production. Plants, particularly oil crops, are a potential renewable and environmentally friendly resource, the NA yield of which can be improved by regulating the biosynthesis pathways of NA via genetic engineering. CgKCS shows promise for engineering high-NA oil for nutraceutical and pharmaceutical applications. For instance, *CgKCS* transgenic *B. carinata* produced oil with up to 44% NA with much reduced EA levels of 5–6% (Taylor et al., 2009), approaching the acceptable level of 5% or less EA, rendering this oil suitable for health application trials (Bettger, 2000). The projected NA yield is 160 g/kg seed in *CgKCS* transgenic *B. carinata*, which is higher than the 115 g/kg seed in *LaKCS* transgenic *B. carinata*, 103 g/kg seed in *CgKCS* transgenic high-EA *B. napus*, and 98 g/kg seed in *T. speciosum*. Furthermore, *LaKCS* transgenic *B. carinata* oil does not meet health requirements due to its high EA contents. In addition to the high seed yields, *CgKCS* transgenic *B. carinata* is the best performer among all the NA seed sources, and its oil has been examined in animal disease model systems for nutraceutical and pharmaceutical applications. In addition, the other VLCFAs, including 18:2, 18:3, and 26:1, totaled 25–30% in the *CgKCS* transgenic *B. carinata* (Taylor et al., 2009). If the NA levels approach 80%, the cost of producing these NAs and their derivatives would be substantially reduced to meet the forecasted increase in demand for NA seed oil products for nutraceutical, pharmaceutical, and chemical industries.

Numerous studies have indicated that the accumulation of NA in seed oil is controlled by complex mechanisms (Guo et al., 2009; Taylor et al., 2009, 2011; Huai et al., 2015). High KCS-specificity and activity during seed development and the ability to assemble NA to more TAG sites are necessary steps for increasing NA contents; however, these steps are not sufficient. Rather, additional genes must be inserted to obtain High Erucic Acid Rapeseed (HEAR) cultivars accumulating seed oil with a homogeneous FA composition. In the future, ideal NA production in seed oils can be obtained via the co-expression of multiple genes, including *KCS* genes that can completely convert substrate to NA, additional genes such as acyltransferase genes that can assemble NA to sn-1,2,3 sites efficiently, and genes that can improve the oil content, in appropriate cruciferous oil crops with enriched substrate, high oil content, and high yield.

## Author Contributions

XZh and FL designed and structured the review. PW and XX collected the information. FL and PW organized the tables. FL and XX prepared the figures. FL and XZh wrote and revised the manuscript. GW, XZh, and XZe commented on the manuscript. All the authors read and approved the final manuscript.

## Conflict of Interest

XZh was employed by Life Science and Technology Center, China National Seed Group Co. Ltd., Wuhan, China. The remaining authors declare that the research was conducted in the absence of any commercial or financial relationships that could be construed as a potential conflict of interest.

## Footnotes

1 http://www.bioriginal.com/products/formats.php
